# Discovery of *Vibrio cholerae* in Urban Sewage in Copenhagen, Denmark

**DOI:** 10.1007/s00248-024-02419-7

**Published:** 2024-07-31

**Authors:** Christian Brinch, Saria Otani, Patrick Munk, Maaike van den Beld, Eelco Franz, Frank M. Aarestrup

**Affiliations:** 1 Research Group for Genomic Epidemiology, Technical University of Denmark, Kgs, Lyngby, Denmark; 2 Centre for Infectious Disease Control, National Institute for Public Health and the Environment, Bilthoven, The Netherlands

**Keywords:** *Vibrio cholerae*, Metagenomic genome recovery, Sewage surveillance, Pathogen detection

## Abstract

We report the discovery of a persistent presence of *Vibrio cholerae* at very low abundance in the inlet of a single wastewater treatment plant in Copenhagen, Denmark at least since 2015. Remarkably, no environmental or locally transmitted clinical case of *V. cholerae* has been reported in Denmark for more than 100 years. We, however, have recovered a near-complete genome out of 115 metagenomic sewage samples taken over the past 8 years, despite the extremely low relative abundance of one *V. cholerae* read out of 500,000 sequenced reads. Due to the very low relative abundance, routine screening of the individual samples did not reveal *V. cholerae*. The recovered genome lacks the gene responsible for cholerae toxin production, but although this strain may not pose an immediate public health risk, our finding illustrates the importance, challenges, and effectiveness of wastewater-based pathogen surveillance.

## Introduction

Metagenomic analysis of sewage offers affordable and rapid surveillance of pathogens in an unbiased and predominantly healthy cross section of a large human population. In recent years, genome sequencing of sewage has been used to monitor the prevalence of SARS-CoV-2 in urban populations [1], the distribution of antimicrobial resistance (AMR) genes across the world [2, 3], and the entire virome [4]. While sewage is easily obtainable, detecting low abundance pathogens, using short-read metagenomic shotgun sequencing, is a challenge. That is because each sample besides fecal matter from thousands of randomly chosen individuals also contains a significant environmental component, as well as material originating from sewer dwelling animals and organic waste material that are spilled into sinks and drains from, e.g., medical facilities, industries, and ordinary households. It is further complicated by the fact that short reads randomly sequenced from a given microorganism might share sequence similarities to several different, not necessarily closely related, microorganisms and thus, be mistakenly assigned. It is well-known that analyses of metagenomic data can result in false-positive detection of pathogens mainly because of reads mapping to conserved regions in genomes [5]. Long-read sequencing makes it considerably easier to correctly assign reads to a species, but the lower read yield and higher error rate make it hard to reliably detect low-abundance organisms.

While *V. cholerae* has previously been reported in and around wastewater treatment plants [6, 7], these reports usually come from areas where cholera is endemic and over a short period of time. Here, we report an in-depth metagenomic analysis of urban sewage from Copenhagen, Denmark, prompted by a positive *Vibrio cholerae* result from routine qPCR screening of a few samples from a single wastewater treatment plant in Copenhagen. We also addressed the limitations of short-read metagenomics to recover a nearly complete *V. cholerae* genome and confirm the qPCR findings, despite its very low but constant relative abundance in the samples. Our finding is unusual because *V. cholerae* naturally occur in salt- or brackish water and is not endemic to northern Europe (CDC Yellow Book 2024), due to sub-optimal growth conditions. Clinical cases of locally transmitted cholera have not been reported in Denmark since the 1850s, but 0–2 travel-related cases are reported annually.

## Materials and Methods

### Sample Collection

Untreated sewage has been collected regularly from three different wastewater treatment plants (BIOFOS Rensesanlæg: Avedøre (RA), Damshusåen (RD), and Lynetten (RL)) in Copenhagen, Denmark since late 2015. In this paper, we consider the subset of samples that were collected at the Avedøre site, which is located approximately 10 km from the city center of Copenhagen, Denmark (55.6086, 12.450). The first batch of samples from 2015 to 2018 were published by Brinch et al. [8], in a study that described the Copenhagen resistome. A second batch of samples obtained between 2019 and 2021 was published by Becsei & Fuschi [9], also with focus on AMR. We include 11 additional samples, eight from the inlet of the RA treatment plant and three upstream samples, collected in 2022 and 2023. They are previously unpublished. The total number of RA samples amounts to 115 plus three from the catchment area. In all cases, 0.5 l of untreated and unfiltered sewage was collected at the inlet to the treatment plant over the course of 24 h using a flow-proportional sampler. The samples were frozen and stored at − 80 °C until DNA extraction.

### Sample Preparation and Sequencing

Samples have been collected over the course of nearly 10 years (Table 1). Both laboratory procedures and sequencing technology have undergone changes over that period of time. Thus, not all samples have been prepared and sequenced using the exact same protocols. For details on the sample preparation and sequencing procedure of the published data, please refer to [8] and [9]. For the unpublished samples, ten sewage samples were prepared like in [2], while one was sequenced using Oxford Nanopore Technology (ONT) with the Ligation kit (LSK114) [10] after DNA extraction using Quick-DNA HMW MagBead kit (Zymo Research, Irvine, USA).

**Table 1:** Overview of the sample sequencing campaign

Batch	Period	Platform	Number of samples	Number of bases [Gbp]	Mean fragment count [millions]	Standard deviation [millions]	Published in
1	09/01/2016–05/03/2017	MiSeq	24	18.50	2.31	1.64	Brinch et al. (2020)
	23/11/2015–18/10/2018	NextSeq 550	31	194.36	23.88	8.33	
	03/02/2016	HiSeq 3000	1	14.02	52.24		
2	20/01/2019–28/09/2020	NovaSeq 6000	51	583.98	39.58	17.93	Becsei & Fuschi et al. (2024)
3	09/11/2022–25/01/2023	NextSeq 550	6	22.04	15.12	7.27	This study
	19/04/2023	NextSeq 550	1	127.31	469.76		
	11/01/2023	Oxford Nanopore	1	4.95	0.25		
Upstream	19/04/2023	NextSeq 550	3	312.33	371.37	160.86	
Total	23/11/2015–19/04/2023		118	1277.49	38.49		

### V. cholerae Detection Using qPCR

As part of a broader study comparing the sewage microbiomes across five European cities, 117 samples from the three sites in Copenhagen were investigated by real-time PCR for the detection of *V. cholerae*. The reaction was conducted in six multiplex reactions of 20 µl volume with SensiFast (Bioline, GC Biotech, Waddinxveen, Netherlands) mastermix, primer and probe (Table 2) concentrations of 0.5 µM and 0.25 µM respectively. PCRs were run on a Lightcycler 480 II instrument (Roche Diagnostics, Basel, Switzerland) using 45 cycles of 5 s of denaturation at 95 °C and 30 s annealing at 60 °C using Phocid herpesvirus as internal control.

**Table 2:** qPCR probe and primer sequences

toxR	*Vibrio cholerae*	Schets et al	*ToxRf*	gtgccttcatcagccactgtag
			*ToxRr*	agcagtcgattccccaagtttg
			*ToxRp*	TexRed-caccgcagccagccaatgtcgt-BHQ1

### Culture-Based Analysis

Sewage samples from the Avedøre site were also tested for *V. cholerae* presence using culture-based techniques [11]. Briefly, sewage samples were enriched in alkaline buffered peptone water (ABPW, pH 8.6 ± 0.2) followed by *Vibrio* selective media culturing on thiosulphate citrate bile sucrose agar (Sigma). Yellow colonies (presumably *V. cholerae* according to the manufacturer’s guidelines) were selected for biochemical identification using API20E system (bioMérieux, Marcy L’Etoile, France). For rapid PCR-based identification, DNA yields were also extracted from the yellow colonies and Avedøre sewage samples, and used for *V. cholerae* detection using PCR amplification of *toxR* and *ctxA* genes as described in [11].

### Bioinformatics Tools

All short-read mapping and alignment were done with KMA version 1.4.11 [12]. Initial metagenomic assembly was done using MEGAHIT version 1.2.9 [13], and after that, all short-read de novo assembly was done with SPAdes version 3.15.5 [14] and Flye version 2.9.3 for long-read assembly [15]. Genome annotation was done with Prokka version 1.11 [16], and genome completeness was estimated with CheckM2 version 0.1.3 [17]. A phylogenetic tree was constructed with CSI phylogeny version 7.31.0 [18]. Metabat2 version 2.12.1 [19] was used to bin the initial metagenomic assembly, and Kraken2 version 2.0.7-beta [20] was used to identify the *V. cholerae* bin. All hand-editing (breaking and patching contigs based on read depth variations and coverage discontinuities), visualization, quality testing, and contig management were done using Geneious Prime (Geneious version 2023.2 created by Biomatters. Available from https://www.geneious.com).

### Recovery of the Copenhagen V. cholerae Genome

Constructing the genome was a long, iterative process which involved many qualitative decisions along the way. We started with the metagenomic co-assembly from Avedøre (RA) previously reported by Becsei & Fuschi [9]. Briefly, this co-assembly was the result of the MEGAHIT assembler which was run on the 51 NovaSeq samples. The resulting contigs were taxonomically assigned using kraken2, and some were assigned to *V. cholerae*. We extracted all these *V. cholerae*-annotated contigs (*n* = 1428) and extended them by aligning all Illumina reads against them, with KMA using a very stringent setting, requiring 95% of the bases in the reads to match (KMA flags: -mrs 0.95 -mrc 0.95). The aligning reads were recovered and re-assembled de novo with SPAdes using the previous set of contigs as “untrusted contigs” (SPAdes flags: –cov-cutoff off –careful –untrusted-contigs). Original MEGAHIT contigs that did not align to a contig in the new SPAdes set of contigs were discarded, thereby weeding out contamination. This process was repeated several times to extend the contigs as much as possible. In some cases, contigs were edited by hand, by breaking and patching contigs based on visual inspection of anomalies and discontinuities in read alignments.

**Fig. 1:**
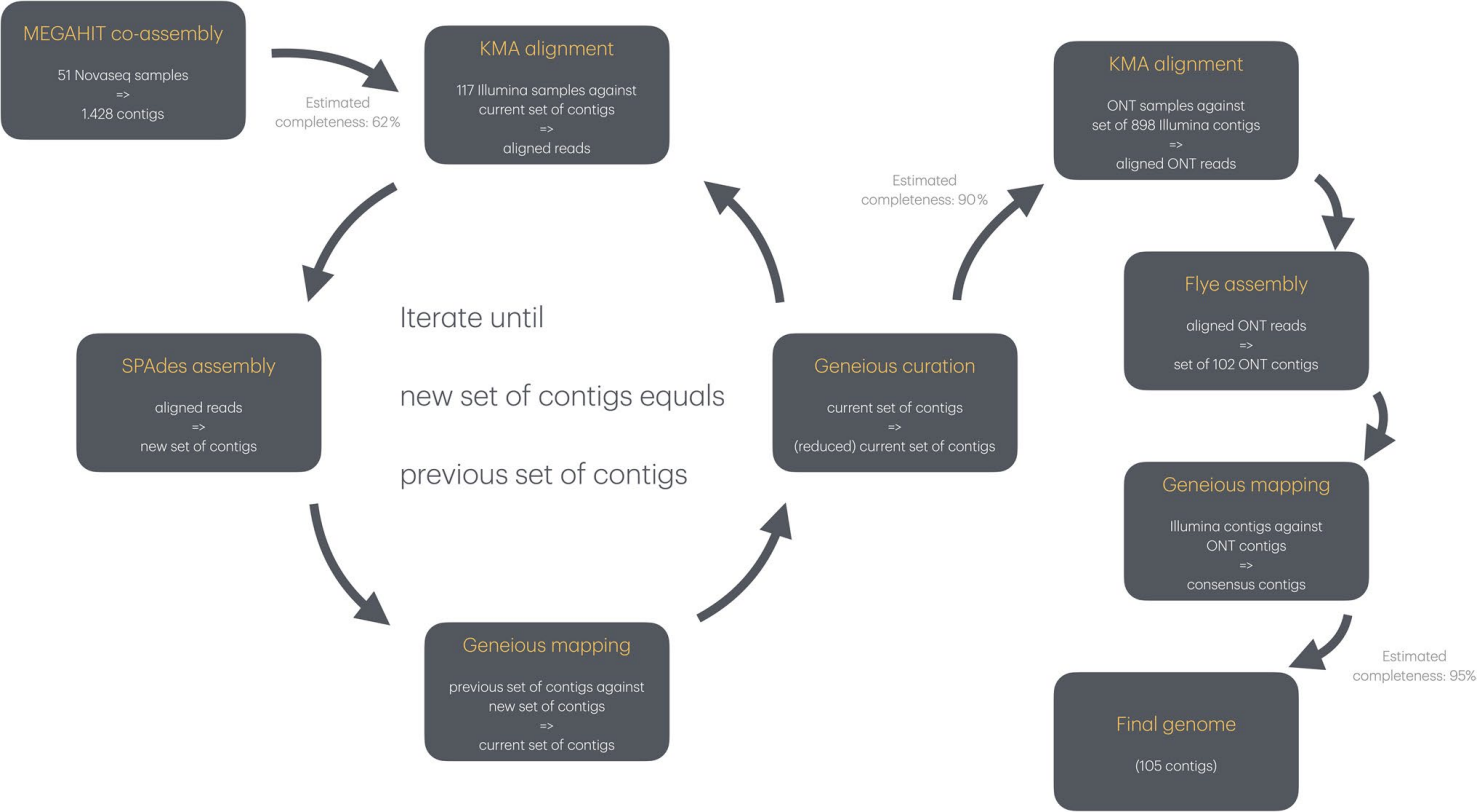
Schematic representation of the metagenomic workflow, resulting in the recovery of a nearly complete *V. cholerae* genome

Eventually, we had 898 contigs that we could not extend any further. When the contigs could not be extended any further, ONT sequences were then aligned against the 898 Illumina contigs, and the aligning ONT reads were assembled using Flye. This resulted in a set of 102 ONT contigs which were polished with the short reads from the Illumina sequenced samples. Three of the 898 Illumina contigs did not align to any of the long-read contigs, so these three contigs were included in the set of contigs that makes up the final genome consisting of 105 contigs (length = 3,577,379, N50 = 71,987). The workflow is graphically represented in Fig 1.

To calculate the relative abundance of *V. cholerae*, we used the CLR transformation, which is defined as the logarithm to the ratio of each part to the geometric mean of parts. We considered the two-part composition, with one part being the count of reads aligning to *V. cholerae* and the other part being the count of reads aligning to any other bacterial genome, i.e., the bacteriome. To determine the bacteriome abundance, we aligned all samples against a curated database of ~25.000 complete bacterial genomes (and no plasmids) downloaded from NCBI. The CLR abundance of *V. cholerae* indicates the extent to which *V. cholerae* is less abundant compared to the overall bacteriome, measured in log-fold. A *V. cholerae* CLR abundance of −6 implies that the CLR abundance of the bacteriome is +6 and this means that *V. cholerae* is e^12^ ≈ 163,000 times less abundant than the bacteriome or, in other words, that out of 163,000 bacterial reads, one read will belong to *V. cholerae*.

## Results and Discussion

A near-complete *V. cholerae* genome was constructed out of 118 metagenomic samples taken over the past 8 years. Before we began a targeted search for *V. cholerae*, *Vibrio* reads were found sporadically and had very low abundances in Copenhagen sewage [5]. Samples from the early part of the sewage surveillance program were sequenced on Illumina MiSeq and NextSeq, obtaining an average of 2 and 20 million paired-end reads per sample, respectively. The shallow sequencing did not constantly produce any alignments to the *V. cholera* 16S rRNA, which was used at the time to quantify the bacterial fraction of the samples. *V. cholerae* was first detected in Copenhagen with traditional qPCR pathogen screening among sewage samples from several European cities and this triggered our targeted metagenomic search. Out of the 117 samples from Copenhagen (all three sites) that were tested with qPCR, 37 were positive for *V. cholerae*, all from the Avedøre site, necessitating further metagenomics investigations, and this prompted a new sequencing campaign. Only one Avedøre sample was qPCR negative, whereas all tested samples from the other two sites were negative. Since about 2019, additional samples have been obtained and sequenced to a greater depth than previously, with up to 30–50 million reads per sample. When these metagenomes were aligned against a bacterial whole genome database (and not just 16S rRNA sequences), they consistently revealed reads aligning to *V. cholerae*, and again only from Avedøre site (RA), in agreement with the qPCR results. The two other Copenhagen sites (RD and RL) were consistently negative for *V. cholerae* using both metagenomics and qPCR approaches. When all samples, including the older ones, were revisited following the positive qPCR results and analyzed using the full bacterial genome database, they were found to have a similar abundance but at much lower count rates, consistent with the lower sequencing depth. Some of the earliest MiSeq samples still had no reads that aligned to *V. cholerae*, but given the low abundance and shallow sequencing, the absence of even a single read was expected. Toward the end of the sampling campaign in April 2023, four samples were sequenced to a significantly higher depth (between 100 and 130 million reads). Three of these samples were taken upstream of the treatment plant (4 – 10 km) from the three main lines leading into the treatment plant inlet, and all were negative for *V. cholerae*.

To confirm our tentative findings, based on qPCR and plain read alignment, several parallel approaches were tested. PCR using standard *V. cholerae* primers of *toxR* and *ctxA* genes were negative, and culturing on selective media only showed *Vibrio metschnikovii* growth (API20E biochemical results). This was probably due to the very low relative abundance of *V. cholerae* in the samples and lack of virulence or toxin genes.

**Fig. 2:**
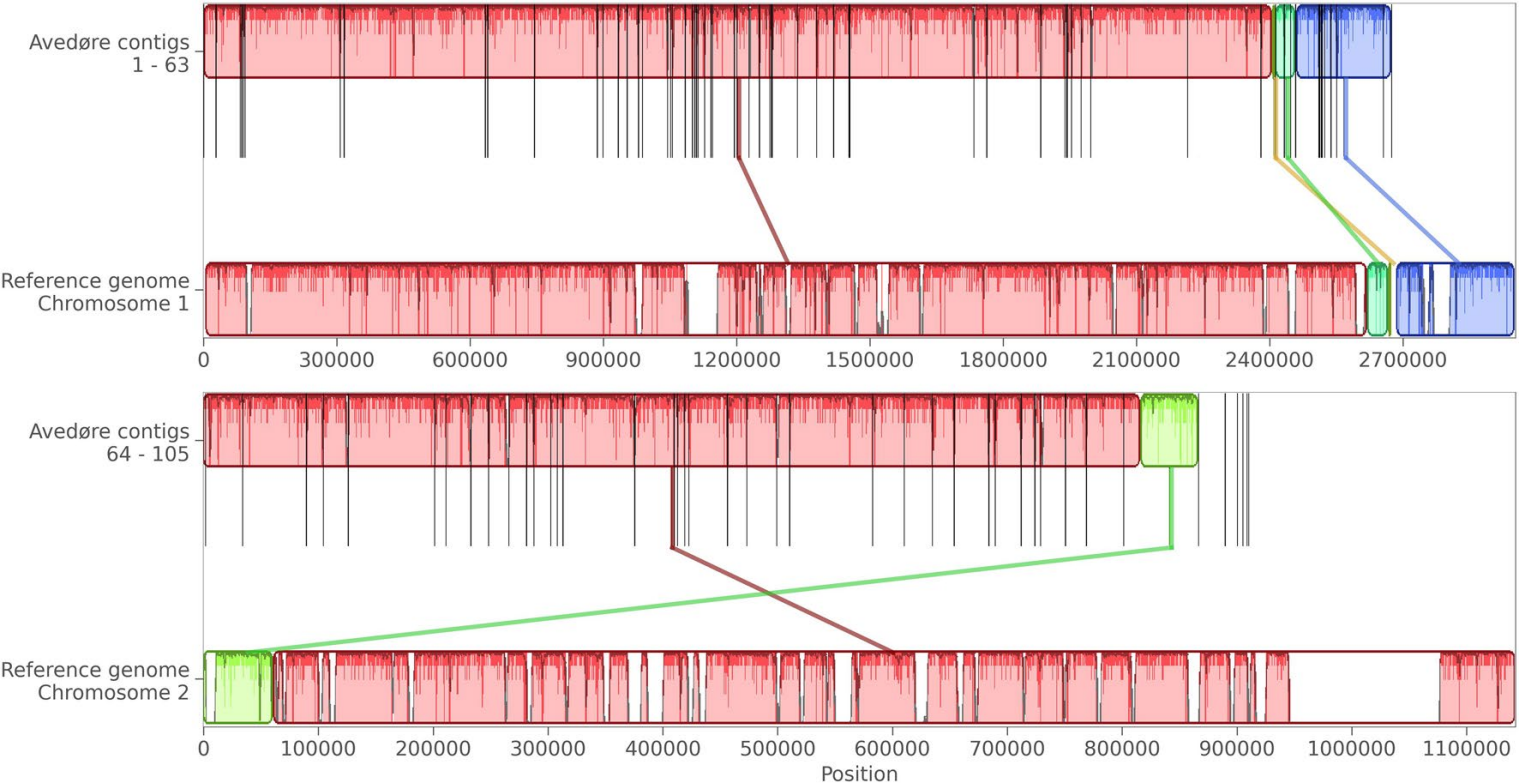
A mauve alignment of our recovered genome against each of the two chromosomes of the *V. cholerae* reference genome

**Fig. 3:**
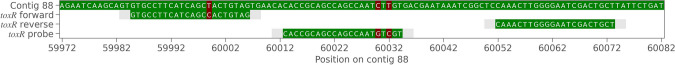
*toxR* primer and probe sequences aligned against contig 88 from the recovered *V. cholerae* genome

One thousand four hundred twenty-eight contigs from the deep co-assembly of the Avedøre site were taxonomically assigned to *V. cholerae*. This set of contigs was estimated to represent a 62% complete and 8% contaminated genome, according to CheckM2. After iteratively extending the length and reducing the number of contigs, we ended up with 898 contigs, which were estimated to be 90% complete and 3% contaminated. Adding the ONT sample further reduced the number of contigs to 102, resulting in a genome that was 95% complete and 0.06% contaminated. Mauve alignment of the final genome against the *V. cholerae* reference genome (Fig. 2, Genome assembly ASM836960v1, [21]) showed that the nearly complete genome could align well. No alignments were found for the cholera toxin *ctxA* gene primers or probe [11], but both primer and probe sequences for the central regulatory protein *toxR* aligned to Contig 88 (positions 59,980 and 60,080 – Fig. 3). Blasting the region around the alignment resulted in a 99.2% grade hit to *toxR* from *V. cholerae*.

**Fig. 4:**
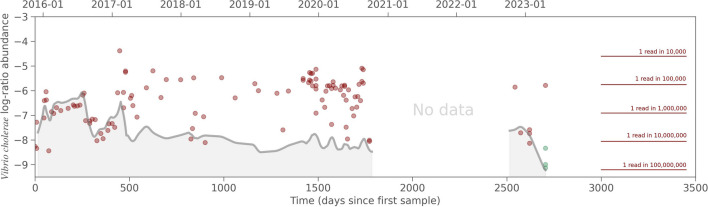
Observed *V. cholerae* abundance (CLR) over time. The red horizontal bars indicate the corresponding proportions as 1 V*. cholerae* read in n bacterial reads. The gray line indicates the noise floor defined as the CLR of 1 read. The noise floor is based on the running average sequence depth. The three points plotted in green are upstream samples

It is difficult to quantify the abundance of rare species in a complex metagenome due to the presence of regions that are conserved across many species. When mapping our reads to the *V. cholerae* contigs, many reads that do not originate from *V. cholerae* will align to these regions, forming depth “towers” with up to a thousand times more depth than the other parts of the genome. It is difficult to determine whether a read that maps to one of these depth towers originates from *V. cholerae* or another unrelated species. Therefore, we statistically adjusted the number of reads in the tower regions based on depth difference between the tower and the baseline read depth. This adjustment was acceptable except when the abundance was extremely low or zero, that is, when *all* tower reads were not from *V. cholerae.* For instance, after depth correction, 2, 2, and 6 reads mapped statistically to the three upstream samples, respectively. These reads all aligned to tower regions, and not a single read was found outside the towers. Hence, we assume that these ten reads were not *V. cholerae* reads and that the upstream samples are truly negative. After depth corrections, we used the read counts to calculate CLR abundances of *V. cholerae* relative to the bacteriome, which measures the fraction of the bacterial reads belonging to *V. cholerae* (Fig. 4). The noise level is defined as the CLR value obtained if exactly one read aligns, given the sequencing depth. Therefore, samples that fall below the gray line in Fig. 4 have zero reads aligning to *V. cholerae*, and those points can therefore be considered upper limits. The median abundance of *V. cholerae* in the 114 Illumina sequenced samples, where *V. cholerae* reads were found, was estimated to be approximately one *V. cholerae* read in 140,000 bacterial reads. On average, 30% of reads across all samples aligned to a bacterial genome, meaning that to find one *V*. *cholerae* read, approximately half a million reads must be sequenced.

Interestingly, we found that even though *V. cholerae* appears to be consistently present throughout the entire sampling period, some individual samples show very low abundance, to the point where we hardly detect it. This could be explained by other bacterial species increasing their abundance significantly for a short period of time, an effect which has been documented in [9]. Such variations can make *V. cholerae* seem to disappear due to the compositional nature of metagenomics.

**Fig. 5:**
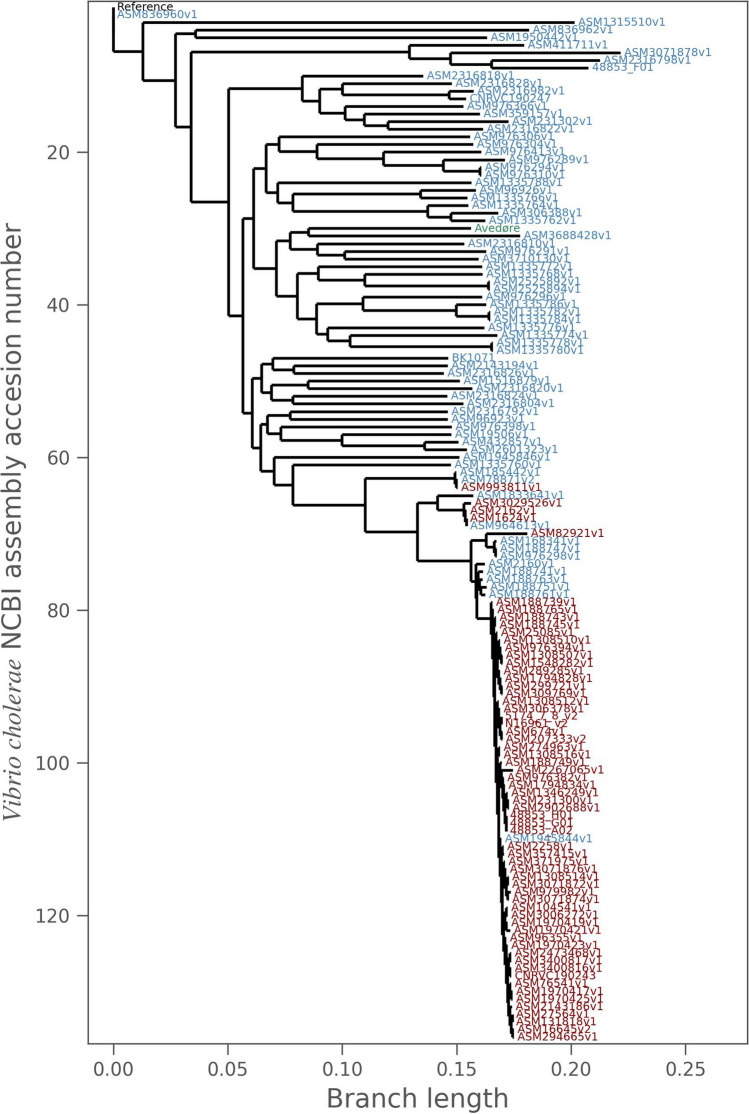
A phylogenetic tree showing the genomic context of our recovered genome. Each leaf represents a complete *V. cholerae* genome. Blue indicates a CTX-negative genome and red indicates a CTX-positive genome. Our genome is shown in green

A phylogenetic tree was constructed to compare our *V. cholerae* genome to the 135 complete *V. cholerae* genomes found on NCBI (at the time of our analysis). The tree was rooted at the reference genome (Fig. 5). The genomes have been colored according to whether they have the *ctxA* gene, where blue is CTX-negative, and red is CTX-positive. Our recovered genome is shown in green, and it does not appear to be special or different when compared to the neighboring genomes. The closest relative is recent and isolated from a human by the CDC [22]*.*

Globally, many initiatives to utilize wastewater for surveillance of potentially all infectious pathogens are underway [23–25]. This includes the possible use of several different methods including qPCR and arrays, but also metagenomics that agnostically will sequence any DNA (or cDNA from RNA) indiscriminately, potentially usable to detect all pathogens including those that are still unknown. This also comes with potential challenges of false-positive signals especially when handling low abundance pathogens. In our case, we originally ignored the first 16S alignments to *V. cholerae* since we also found many reads clearly aligning to conserved “tower” regions. The actual presence was only detectable when combining data from many samples. Thus, for future sewage-based surveillance using metagenomics, it will probably, in most cases, be necessary to establish site-specific signatures as a baseline to which subsequent data can be compared and thus improve the sensitivity and specificity of the surveillance.

Our finding of non-toxigenic but constantly colonizing *V. cholerae* in urban sewage at the inlet of a single site, but not further upstream, also shows that *V. cholerae* can establish itself in environments with an average annual temperature of 9 °C. This also has implications for future reservoirs and potential transmission of *V. cholerae* since more frequent heavy rainfalls are expected in the future resulting in more frequent floodings. In our case, the specific strain was CTX-negative, but this might not always be the case. Theoretically, the presence of *V. cholerae* could be explained by a constant source, delivering the bacteria to the sewer, rather than having it colonizing the sewer itself. This scenario, however, is very unlikely given how stable the abundance of *V. cholerae* is over time and how much the fecal relative contribution to sewage fluctuates from week to week [9].

It is normally assumed that *V. cholerae* do not grow as planktonic cells in the environment, and several studies have suggested chitin rich aquatic fauna as a reservoir [26, 27]. Our study, however, could indicate that cyanobacteria might be a potential reservoir of *V. cholerae* [28], since different cyanobacteria are highly abundant in the sewer system while copepods are not.

Besides the biological observation, our study also illustrates the challenges of using metagenomic sequencing for detection of pathogens. In our case, we originally ignored true low abundance signals because they were masked as false-positive reads; and further investigations, utilizing deep co-assemblies of many metagenomes and contig screening, were not initiated until prompted by a positive qPCR result.

## Data Availability

Sequencing reads are available from ENA under project numbers PRJEB34633, PRJEB68319, and PRJEB76456. The genome is available from NCBI under BioProject number PRJNA1121193. Code, scripts, and pipelines are available upon request.

## References

[1] Crits-Christoph A et al (2021) Genome sequencing of sewage detects regionally prevalent SARS-CoV-2 variants. mBio 12(1):1–9. 10.1128/MBIO.02703-20/ASSET/1FCC5B74-F52F-4B47-82D7-4EC4764697A2/ASSETS/GRAPHIC/MBIO.02703-20-F0003.JPEG 10.1128/MBIO.02703-20/ASSET/1FCC5B74-F52F-4B47-82D7-4EC4764697A2/ASSETS/GRAPHIC/MBIO.02703-20-F0003.JPEG PMC7845645 33468686

[2] Hendriksen RS et al (2019) Global monitoring of antimicrobial resistance based on metagenomics analyses of urban sewage. Nat Commun 10(1):1124. 10.1038/S41467-019-08853-3 30850636 10.1038/S41467-019-08853-3 PMC6408512

[3] Munk P et al (2022) Genomic analysis of sewage from 101 countries reveals global landscape of antimicrobial resistance. Nat Commun 13(1):1–16. 10.1038/s41467-022-34312-7 36456547 10.1038/s41467-022-34312-7 PMC9715550

[4] Fernandez-Cassi X et al (2018) Metagenomics for the study of viruses in urban sewage as a tool for public health surveillance. Sci Total Environ 618:870–880. 10.1016/J.SCITOTENV.2017.08.249 29108696 10.1016/J.SCITOTENV.2017.08.249

[5] Ye SH, Siddle KJ, Park DJ, Sabeti PC (2019) Benchmarking metagenomics tools for taxonomic classification. Cell 178(4):779–794. 10.1016/J.CELL.2019.07.010 31398336 10.1016/J.CELL.2019.07.010 PMC6716367

[6] Gatti M, Stampi S, Donati M, De Luca G, Aschbacher R, Zanetti F (1997) Characteristics of non-O1 Vibrio cholerae isolated from the effluents of a treatment plant. New Microbiol 20(4):311–318 9385600

[7] Okoh AI, Sibanda T, Nongogo V, Adefisoye M, Olayemi OO, Nontongana N (2015) Prevalence and characterisation of non-cholerae Vibrio spp. in final effluents of wastewater treatment facilities in two districts of the Eastern Cape Province of South Africa: implications for public health. Environ Sci Pollut Res Int 22(3):2008–2017. 10.1007/s11356-014-3461-z 25167817 10.1007/s11356-014-3461-z PMC4308643

[8] Brinch C, Leekitcharoenphon P, Duarte ASR, Svendsen CA, Jensen JD, Aarestrup FM (2020) Long-term temporal stability of the resistome in sewage from Copenhagen. mSystems 5(5):10–128. 10.1128/MSYSTEMS.00841-20 10.1128/MSYSTEMS.00841-20 PMC7577296 33082278

[9] Becsei Á, Fuschi A, Otani S, Kant R, Weinstein I, Alba P, Stéger J, Visontai D, Brinch C, de Graaf M, Schapendonk CME, Battisti A, De Cesare A, Oliveri C, Troja F, Sironen T, Vapalahti O, Pasquali F, Bányai K, Makó M, Pollner P, Merlotti A, Koopmans M, Csabai I, Remondini D, Aarestrup FM, Munk P (2024) Time-series sewage metagenomics can separate the seasonal, human-derived and environmental microbial communities, holding promise for source-attributed surveillance. bioRxiv, pp 2024–05. 10.1101/2024.05.30.596588

[10] Gand M et al (2024) Towards facilitated interpretation of shotgun metagenomics long-read sequencing data analyzed with KMA for the detection of bacterial pathogens and their antimicrobial resistance genes. Front Microbiol 15:1336532. 10.3389/FMICB.2024.1336532/BIBTEX 38659981 10.3389/FMICB.2024.1336532/BIBTEX PMC11042533

[11] Schets FM, van den Berg HHJL, Marchese A, Garbom S, de Roda Husman AM (2011) Potentially human pathogenic vibrios in marine and fresh bathing waters related to environmental conditions and disease outcome. Int J Hyg Environ Health 214(5):399–406. 10.1016/J.IJHEH.2011.05.003 10.1016/j.ijheh.2011.05.003 21664866

[12] Clausen PTLC, Aarestrup FM, Lund O (2018) Rapid and precise alignment of raw reads against redundant databases with KMA. BMC Bioinformatics 19(1):1–9. 10.1186/S12859-018-2336-6 30157759 10.1186/S12859-018-2336-6 PMC6116485

[13] Li D, Liu C-M, Luo R, Sadakane K, Lam T-W (2015) MEGAHIT: an ultra-fast single-node solution for large and complex metagenomics assembly via succinct *de Bruijn* graph. Bioinformatics 31(10):1674–1676. 10.1093/bioinformatics/btv033 25609793 10.1093/bioinformatics/btv033

[14] Prjibelski A, Antipov D, Meleshko D, Lapidus A, Korobeynikov A (2020) Using SPAdes De Novo Assembler. Curr Protoc Bioinforma 70(1):e102. 10.1002/CPBI.102 10.1002/CPBI.102 32559359

[15] Kolmogorov M, Yuan J, Lin Y, Pevzner PA (2019) Assembly of long, error-prone reads using repeat graphs. Nat Biotechnol 37(5):540–546. 10.1038/s41587-019-0072-8 30936562 10.1038/s41587-019-0072-8

[16] Seemann T (2014) Prokka: rapid prokaryotic genome annotation. Bioinforma Oxf Engl 30(14):2068–2069. 10.1093/BIOINFORMATICS/BTU153 10.1093/BIOINFORMATICS/BTU153 24642063

[17] Chklovski A, Parks DH, Woodcroft BJ, Tyson GW (2023) CheckM2: a rapid, scalable and accurate tool for assessing microbial genome quality using machine learning. Nat Methods 20(8):1203–1212. 10.1038/s41592-023-01940-w 37500759 10.1038/s41592-023-01940-w

[18] Kaas RS, Leekitcharoenphon P, Aarestrup FM, Lund O (2014) Solving the problem of comparing whole bacterial genomes across different sequencing platforms. PLoS ONE 9(8):e104984. 10.1371/JOURNAL.PONE.0104984 25110940 10.1371/JOURNAL.PONE.0104984 PMC4128722

[19] Kang DD et al (2019) MetaBAT 2: An adaptive binning algorithm for robust and efficient genome reconstruction from metagenome assemblies. PeerJ 7:2019. 10.7717/PEERJ.7359/SUPP-3 10.7717/PEERJ.7359/SUPP-3 PMC6662567 31388474

[20] Wood DE, Lu J, Langmead B (2019) Improved metagenomic analysis with Kraken 2. Genome Biol 20(1):1–13. 10.1186/S13059-019-1891-0/FIGURES/2 31779668 10.1186/S13059-019-1891-0/FIGURES/2 PMC6883579

[21] Bina RF, Bina JE, Weng Y (2022) Genome sequence of Vibrio cholerae strain RFB16, isolated from North Park Lake in Allegheny County, Pennsylvania. Microbiol Resour Announc 9(10):10–128. 10.1128/MRA.00111-20 10.1128/MRA.00111-20 PMC7171212 32139572

[22] Liang KYH et al (2020) A Vibrio cholerae core genome multilocus sequence typing scheme to facilitate the epidemiological study of cholera. J Bacteriol 202(24):10–128. 10.1128/JB.00086-20 10.1128/JB.00086-20 PMC7685551 32540931

[23] Diamond MB et al (2022) Wastewater surveillance of pathogens can inform public health responses. Nat Med 28(10):1992–1995. 10.1038/s41591-022-01940-x 36076085 10.1038/s41591-022-01940-x

[24] Levy JI, Andersen KG, Knight R, Karthikeyan S (2023) Wastewater surveillance for public health. Science 379(6627):26–27. 10.1126/SCIENCE.ADE2503/ASSET/458E22C4-08E8-457E-8278-1C52CE613922/ASSETS/IMAGES/LARGE/SCIENCE.ADE2503-F1.JPG 36603089 10.1126/SCIENCE.ADE2503/ASSET/458E22C4-08E8-457E-8278-1C52CE613922/ASSETS/IMAGES/LARGE/SCIENCE.ADE2503-F1.JPG PMC10065025

[25] Aarestrup FM, Woolhouse MEJ (2020) Using sewage for surveillance of antimicrobial resistance. Science 367(6478):630–632. 10.1126/SCIENCE.ABA3432/ASSET/6F36E8AC-9D21-46C2-98E4-8E5016BB8C7E/ASSETS/GRAPHIC/367_630_F2.JPEG 32029617 10.1126/SCIENCE.ABA3432/ASSET/6F36E8AC-9D21-46C2-98E4-8E5016BB8C7E/ASSETS/GRAPHIC/367_630_F2.JPEG

[26] Sun S, Xiang Q, Tay M, Kjelleberg S, Rice SA, Mcdougald D (2015) Quorum sensing-regulated chitin metabolism provides grazing resistance to Vibrio cholerae biofilms. ISME J 9:1812–1820. 10.1038/ismej.2014.265 25615438 10.1038/ismej.2014.265 PMC4511936

[27] Hayes CA, Dalia TN, Dalia AB (2017) Systematic genetic dissection of chitin degradation and uptake in Vibrio cholerae. Environ Microbiol 19(10):4154–4163. 10.1111/1462-2920.13866 28752963 10.1111/1462-2920.13866 PMC5647239

[28] Islam MS, Zaman MH, Islam MS, Ahmed N, Clemens JD (2020) Environmental reservoirs of Vibrio cholerae. Vaccine 38(Suppl 1):A52–A62. 10.1016/J.VACCINE.2019.06.033 31285087 10.1016/J.VACCINE.2019.06.033

